# Association between serum vitamin D deficiency and visceral fat indices in adolescents: The Ewha Birth and growth cohort study

**DOI:** 10.1371/journal.pone.0335507

**Published:** 2025-10-31

**Authors:** Hyelim Lee, Hyunjin Park, Seunghee Jun, Hyeseung Jang, Young Sun Hong, Kyunghee Jung-Choi, Hye Ah Lee, Hyesook Park

**Affiliations:** 1 Department of Preventive Medicine, College of Medicine, Ewha Womans University, Seoul, Republic of Korea; 2 Graduate program in System Health Science and Engineering, Ewha Womans University, Seoul, Republic of Korea; 3 Department of Internal Medicine, College of Medicine, Ewha Womans University, Seoul, Republic of Korea; 4 Department of Occupational and Environmental Medicine, College of Medicine, Ewha Womans University, Seoul, Republic of Korea; 5 Clinical Trial Center, Ewha Womans University Mokdong Hospital, Seoul, Republic of Korea; University of Diyala College of Medicine, IRAQ

## Abstract

**Purpose:**

Vitamin D plays a crucial role in cardiometabolic health, but its association with visceral fat in adolescents remains unclear. This study aimed to evaluate the relationship between vitamin D levels visceral fat indices—Hypertriglyceridemic Waist Phenotype (HWP), Visceral Adiposity Index (VAI), and Lipid Accumulation Product (LAP)—which serve as practical markers for visceral fat.

**Methods:**

This study analyzed 238 adolescents (aged 13–15) data from the Ewha Birth and Growth Study, a longitudinal Korean cohort. Serum 25-hydroxyvitamin D [25(OH)D] levels were measured and categorized as deficiency (<20 ng/mL) or non-deficiency (≥20 ng/mL). Visceral fat was assessed using HWP, VAI, and LAP as surrogate markers for visceral adiposity. Logistic and linear regression analyses were performed to evaluate associations, adjusting for key covariates. Sensitivity analyses used alternative HWP definitions and vitamin D categorizations.

**Results:**

Among 238 adolescents, 76.0% had vitamin D deficiency, and 7.1% had HWP. Logistic regression indicated a lower HWP risk in the non-deficiency group with a borderline level of significance (*p* = 0.060). Sensitivity analyses confirmed a significantly lower HWP risk in the non-deficiency group under the lowered TG criterion and also showed that, in the three-category classification of vitamin D status, the non-deficiency group had a significantly lower HWP risk than the severe deficiency group, with a decreasing trend as vitamin D levels increased. Multiple linear regression showed inverse associations between vitamin D levels and log VAI (*β* = –0.020, *p* = 0.008) and log LAP (*β *= –0.018, *p* = 0.060).

**Conclusions:**

The inverse relationship observed between vitamin D levels and visceral fat indices suggests a potential role in adiposity regulation and cardiometabolic health. Enhancing vitamin D status may help prevent obesity and reduce cardiometabolic risks in adolescents.

## Introduction

Vitamin D is an essential nutrient and hormone regulating calcium-phosphorus metabolism for bone health and plays a key role in immunity, cell growth, differentiation, and other physiological processes [1]. Growing evidence suggests that vitamin D deficiency is associated with a wide range of adverse health conditions, including cardiometabolic diseases, as well as musculoskeletal and mental illnesses. Given its widespread prevalence and health implications, vitamin D deficiency is now recognized as a critical global public health issue [2–4].

A comprehensive 2025 meta-analysis of 586 studies across 102 countries reported that about 75% of the population had vitamin D insufficiency or deficiency (<30 ng/mL), with mean levels slightly lower in children than in adults [5]. In Korea, data from 332 medical institutions revealed that 79.8% of boys and 83.8% of girls aged 0–18 years were vitamin D insufficient or deficient, with rates peaking at about 96% during adolescence [6]. Consistently, the Korea National Health and Nutrition Examination Survey (KNHANES) confirmed that adolescents aged 10–19 years exhibited the highest prevalence of vitamin D insufficiency and deficiency among all age groups [7]. Despite typically being a period of greater outdoor activity and sun exposure, adolescents show high rates of vitamin D deficiency. This suggests that shifts in lifestyle, dietary habits, and limited sun exposure may be contributing factors.

Beyond vitamin D deficiency, the prevalence of obesity, abdominal obesity, and prediabetes among Korean adolescents has nearly doubled over the past decade, while metabolic syndrome and hypertension have continued to rise, suggesting an increasing burden of cardiometabolic diseases [8–10]. As these risks often persist into adulthood, early intervention during adolescence is crucial for reducing long-term health complications.

Cardiometabolic health is a major public health concern, with abdominal obesity playing a critical role in its pathogenesis. Visceral fat contributes to cardiometabolic dysfunction through the secretion of inflammatory mediators and adipokines [11]. Vitamin D may help counteract these effects by enhancing energy expenditure, reducing visceral fat accumulation, and exerting anti-inflammatory and lipid-modulating properties. In addition, it may influence adiposity regulation through pathways such as PPAR-γ signaling. Through these mechanisms, it may support cardiometabolic health and reduce the risk of cardiometabolic diseases [12,13]. Conversely, obesity itself may also contribute to vitamin D deficiency through reduced physical activity and sequestration of vitamin D in adipose tissue, suggesting a potential bidirectional relationship between vitamin D and visceral fat [13].

Visceral fat can be measured with MRI and CT as the gold standard, but cost and radiation exposure limit their use in epidemiologic studies. In addition, body composition analysis (BIA), which can estimate visceral adipose tissue (VAT) area, is difficult to perform in unequipped settings, so surrogate markers such as Hypertriglyceridemic Waist Phenotype (HWP), Visceral Adiposity Index (VAI), and Lipid Accumulation Product (LAP) using blood tests and basic anthropometry are utilized.

HWP, defined by waist circumference and triglyceride levels, is a predictor of the atherogenic metabolic triad, characterized by hyperinsulinemia, elevated apolipoprotein B, and small Low-Density Lipoprotein (LDL) particles [14–16]. VAI incorporates waist circumference (WC), triglycerides (TG), HDL cholesterol, and BMI, through sex-specific equations to assess visceral fat accumulation [17]. Similarly, LAP is derived from WC and TG and is calculated through sex-specific equations, serving as an indicator of lipid accumulation [18]. These indices are used as surrogate markers for visceral fat in epidemiological studies and used for early cardiometabolic risk assessment [16–18]. Adolescence is a critical period when cardiometabolic risk factors begin to emerge, emphasizing the need for early assessment for long-term disease prevention [19]. In epidemiological research, visceral fat indices are valuable tools for assessing cardiometabolic risk in adolescents. Studies have shown that HWP effectively predicts cardiovascular risk factor clustering (area under the curve [AUC]=0.78), VAI identifies metabolically unhealthy phenotypes (AUC = 0.76–0.83), and LAP is a strong predictor of metabolic syndrome (AUC = 0.94) [20–22].

Most studies examining the association between vitamin D and visceral fat indices have focused on adults. In these populations, vitamin D deficiency has been linked to a 3.9-fold increased risk of HWP compared to vitamin D sufficiency [23]. However, findings regarding VAI and LAP remain inconsistent. While one study suggested a positive association between vitamin D levels and LAP, another study conducted in individuals with obesity or diabetes reported an inverse relationship [24,25].

To the best of our knowledge, no studies have specifically examined the relationship between vitamin D status and visceral fat indices in adolescents. Understanding this relationship is particularly important because adolescence represents a critical period for metabolic and hormonal changes, which influence long-term metabolic health [26]. If vitamin D plays a role in regulating visceral fat accumulation during this formative stage, its effects may persist beyond adolescence, influencing the risk of cardiometabolic diseases later in life. Investigating this relationship is essential for developing early intervention strategies to reduce future cardiometabolic risks. Thus, this study aims to evaluate the association between vitamin D levels and three visceral fat indices (HWP, VAI, and LAP) in adolescents.

## Materials and methods

### Study participants

This study utilized data from the Ewha Birth and Growth Study, a longitudinal cohort established with pregnant women who visited Ewha Womans University Mokdong Hospital for prenatal care between 24–28 weeks of gestation from September 2001 to June 2006. A total of 940 children born to participating mothers were enrolled and followed from age 3, with follow-up assessments at ages 5, 7, and annually thereafter. Follow-up is ongoing, and data collection at age 19 is currently in progress. These assessments included anthropometric measurements, dietary surveys, questionnaires, and biological sample collection. Detailed cohort information is available in a previous publication [27].

In this study, we analyzed data from 248 participants who were followed up at ages 13–15. Data were collected between April 2015 and August 2020—from April to May during 2015–2019, and from July to August in 2020. Among them, 10 participants were excluded because of missing data on serum vitamin D levels and key variables required to calculate visceral fat indices (HWP, VAI, LAP), including TG, WC, BMI. Consequently, 238 participants were included in the final analysis.

During follow-up, all participants and their guardians were provided with a comprehensive explanation of the study, and written informed consent was obtained. This study was approved by the Institutional Review Board of Ewha Womans University Seoul Hospital (IRB No. SEUMC 2019-04-035). The dataset used in this analysis was accessed and analyzed in June 2024, and all data were completely anonymous before author access and analysis.

### Anthropometric and blood biomarkers

All anthropometric measurements were obtained by trained examiners. Height and weight were measured to the nearest 0.1 cm and 0.1 kg, respectively, with participants wearing light clothing and barefoot. BMI was calculated as weight (kg) divided by height squared (m^2^). WC (cm) was measured at the narrowest circumference between the iliac crest and the lowest rib using a non-elastic tape. Blood samples were collected following a minimum of 8 hours of fasting. Venous blood was drawn into a 15 mL vacuum-sealed tube, centrifuged, and immediately analyzed for TG and HDL-C using an automated biochemical analyzer (Olympus AU2700; Beckman Coulter Inc., Fullerton, CA, USA).

### Measurement of vitamin D

Serum 25-hydroxyvitamin D [25(OH)D], a key indicator of vitamin D status, was analyzed using stored previously frozen serum samples. Measurements were performed using a chemiluminescent immunoassay (CLIA) on a DxI 800 analyzer (Beckman Coulter, Brea, CA, USA) (intra-assay coefficient of variation ranged from 6.2% to 9.1%). According to the Endocrine Society guidelines, vitamin D levels are classified as deficiency (≤20 ng/mL), insufficiency (20–30 ng/mL), and sufficiency (≥30 ng/mL). However, given that only three participants met the sufficiency criteria, our study categorized participants into two groups based on vitamin D levels: deficiency (<20 ng/mL) and non-deficiency (≥20 ng/mL) [28–30].

### Definition of visceral fat indices

This study assessed visceral fat using three indices: HWP, VAI, and LAP. TG and HDL-C levels were originally measured in mg/dL and converted to mmol/L using standardized conversion formulas:


$$TG(mmol/L)=\frac{TG(mg/dL)}{\text{8}\text{8}.\text{5}\text{7}}$$

$$HDL-C(mmol/L)=\frac{HDL-C(mg/dL)}{\text{3}\text{8}.\text{6}\text{7}}$$

HWP was defined as the simultaneous presence of abdominal obesity and hypertriglyceridemia. Abdominal obesity was determined based on WC at or above the 75^th^ percentile for age and sex, according to the 2007 Korean Children and Adolescents Growth Standards by the Korea Disease Control and Prevention Agency (KDCA) [31]. Hypertriglyceridemia was defined as TG levels ≥130 mg/dL, based on the Korean Pediatric Society guidelines [32]. In this study, individuals satisfying both criteria were classified as having HWP [33]. Individuals with HWP are considered to have a higher risk of developing cardiometabolic diseases.

VAI was calculated using WC, TG, HDL-C, and BMI according to the following sex-specific formulas:


$$BoyVAI=\frac{WC(cm)}{\text{3}\text{9}.\text{6}\text{8}+(\text{1}.\text{8}\text{8}\times BMI)}\times \frac{TG(mmol/L)}{\text{1}.\text{0}\text{3}}\times \frac{\text{1}.\text{3}\text{1}}{HDL-C(mmol/L)}$$

$$GirlVAI=\frac{WC(cm)}{\text{3}\text{6}.\text{5}\text{8}+(\text{1}.\text{9}\text{8}\times BMI)}\times \frac{TG(mmol/L)}{\text{0}.\text{8}\text{1}}\times \frac{\text{1}.\text{5}\text{2}}{HDL-C(mmol/L)}$$

LAP was calculated based on WC and TG levels using the following sex-specific formulas:


BoyLAP=(WC(cm)−65)×TG(mmol/L)

GirlLAP=(WC(cm)−58)×TG(mmol/L)

Higher values of both VAI and LAP indicate greater visceral fat accumulation, which is associated with an increased risk of cardiometabolic diseases. Although VAI and LAP were originally developed for adults, emerging evidence suggests their applicability in assessing metabolic syndrome and cardiovascular risk in children and adolescents [21,22].

### Covariates

Sex, household income, frequency of moderate-intensity physical activity, total energy intake, follow-up month, and use of growth-related dietary supplements were included as covariates using data collected at ages 13–15. These variables were selected based on previous literature [23]. Household income was categorized into three groups: < 3 million South Korean won (KRW), 3–5 million KRW, and ≥5 million KRW. The frequency of moderate-intensity physical activity was classified into three groups: none, 1–2 days per week, and ≥3 days per week. The follow-up month was categorized into April, May, and July–August (July was combined with August due to the small number of participants in July). The use of growth-related dietary supplements was categorized as ‘yes’ or ‘no’.

### Statistical analysis

All statistical analyses were conducted using SAS software (version 9.4; SAS Institute, Cary, NC, USA), with *p* < 0.05 considered statistically significant. Descriptive statistics were presented as mean ± standard deviation for normally distributed variables, median (interquartile range) for non-normally distributed variables, and frequencies (percentages) for categorical variables. TG, VAI, and LAP, which showed non-normal distributions, were log-transformed before analysis. The association between HWP and vitamin D status was assessed using the chi-square test, with Fisher’s exact test applied when expected frequencies were < 5. Logistic regression was performed with vitamin D deficiency (<20 ng/mL) as the reference group, and results were reported as odds ratios (ORs) with 95% confidence intervals (CIs). In cases of rare events, Firth’s logistic regression was utilized to adjust for the small sample bias. For VAI, values exceeding 1.5 times Interquartile Range (IQR) were considered outliers, leading to the exclusion of one participant. Since the LAP values included negative numbers (minimum = –5.4), a constant of 6 was added to all values to ensure positivity before log-transformation. Group differences in visceral fat indices according to vitamin D status were evaluated using the independent t-test for normally distributed variables and the Mann–Whitney U test for non-normally distributed variables. The linear associations between VAI, LAP, and serum 25(OH)D concentration were assessed using linear regression, with results reported beta coefficients (*β*), standard errors (SEs), and *p*-values. Multivariate analyses adjusted for sex, household income, physical activity, total energy intake, and growth-related dietary supplement use.

### Sensitivity analysis

Sensitivity analyses were conducted to assess the robustness of the findings using alternative definitions of vitamin D status and HWP classification. To evaluate whether different categorizations of vitamin D levels influence the observed associations, participants were additionally classified into three groups: non-deficiency (≥20 ng/mL), deficiency (12–19 ng/mL), and severe deficiency (<12 ng/mL).

To further assess the robustness of the association between vitamin D status and HWP, different criteria were used to define HWP:

1) Borderline definition: HWP was defined as WC at or above the 75th percentile and TG ≥ 90 mg/dL [32–33].
2) Conservative definition: HWP was defined as WC at or above the 90th percentile and TG ≥ 130 mg/dL [34].

Based on these criteria, the prevalence of HWP was compared across vitamin D groups, and the association between HWP and vitamin D status was evaluated using logistic regression. Logistic regression was performed using the severe deficiency group as the reference, and the trend test was applied to assess trends across categories.

## Results

### General characteristics of study participants

A total of 238 participants were included, comprising 116 boys (48.7%) and 122 girls (51.3%). Vitamin D deficiency (<20 ng/mL) was observed in 76.0% of participants, with a significantly higher prevalence in girls than in boys (*p* = 0.005). HWP was present in 7.1% of participants, with no significant sex difference (*p* = 0.886). Its components, abdominal obesity (27.3%) and high triglyceride levels (9.7%), also showed no significant sex differences. Both LAP and VAI showed significantly higher median values in girls than boys (*p* = 0.001 and *p* < 0.001, respectively), indicating sex differences (Table 1). Covariate distributions across vitamin D status groups are shown in S1 Table. In the deficiency group, the proportion of participants reporting almost never engaging in moderate physical activity was higher (*p* = 0.023; S1 Table), and the remaining covariates did not show significant differences.

**Table 1:** Characteristics of Study Participants.

Variables	Total (n = 238)	Boys (n = 116)	Girls (n = 122)	*p-*value^a^
Age (years)	13.25 ± 0.56	13.21 ± 0.51	13.28 ± 0.61	0.272
WC (cm)	70.31 ± 8.85	72.67 ± 10.14	68.04 ± 6.77	<0.001
TG (mg/dL)^b^	66.0 (51.0-88.0)	60.5 (45.5-85.0)	69.0 (54.0-90.0)	0.026^c^
BMI (kg/m^2^)	20.63 ± 3.26	20.88 ± 3.63	20.40 ± 2.88	0.260
HDL–C (mg/dL)	51.56 ± 9.74	51.62 ± 9.12	51.57 ± 10.33	0.965
Vitamin D Status				
Serum 25(OH)D (ng/mL)	16.34 ± 5.04	17.53 ± 5.28	15.20 ± 4.53	<0.001
Non-Deficiency, n (%)	57 (24.0)	37 (31.9)	20 (16.4)	0.005
Deficiency, n (%)	181 (76.0)	79 (68.1)	102 (83.6)	
Visceral Fat Indices				
HWP, n (%)	17 (7.1)	8 (6.9)	9 (7.4)	0.886
Abdominal obesity, n (%)	65 (27.3)	29 (25.0)	36 (29.5)	0.435
High TG, n (%)	23 (9.7)	12 (10.3)	11 (9.0)	0.729
LAP^b^	11.80 (7.93-17.53)	9.41 (6.00-16.40)	13.35 (9.60-19.18)	0.001^c^
VAI^b^	0.79 (0.55-0.12)	0.60 (0.43-0.90)	0.97 (0.73-1.42)	<0.001^c^

WC, waist circumference; TG, triglyceride; BMI, body mass index; HDL-C, high-density lipoprotein cholesterol; HWP, hypertriglyceridemic waist phenotype; LAP, lipid accumulation product; VAI, visceral adiposity index. Serum 25-hydroxyvitamin D [25(OH)D] was categorized as Non-deficiency (≥20 ng/mL) and Deficiency (<20 ng/mL). HWP was defined as waist circumference ≥75th percentile and triglycerides ≥130 mg/dL. Abdominal obesity: waist circumference ≥75th percentile; High TG: triglycerides ≥130 mg/dL.

^a^Continuous variables were analyzed using the t-test (normal distribution) or Mann-Whitney U test (non-normal distribution). Categorical variables were analyzed using the chi-square test.

^b^TG, LAP, and VAI are presented as median (interquartile range) due to non-normal distribution. LAP and VAI were analyzed in 237 participants after excluding one outlier. A constant of 6 was added to LAP to address negative values. ^c^ *p*-values for TG, LAP, and VAI were calculated using the Mann–Whitney U test.

### HWP Prevalence by vitamin D status

**Fig 1:**
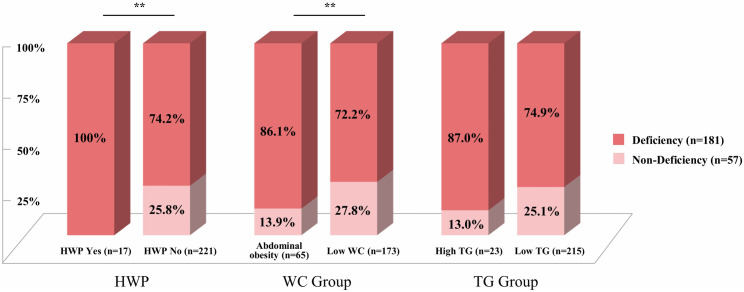
Distribution of Hypertriglyceridemic Waist Phenotype, abdominal obesity, and high triglyceride by serum vitamin D Status. Vitamin D status was defined as Deficiency (<20 ng/mL) and Non-deficiency (≥20 ng/mL) based on serum 25(OH)D concentration. HWP was defined as Abdominal obesity (WC ≥ 75th percentile) and High TG (TG ≥ 130 mg/dL). Based on these criteria, WC was categorized into two groups: Abdominal obesity (WC ≥ 75th percentile) and Low WC (WC < 75th percentile), and TG was categorized into two groups: High TG (TG ≥ 130 mg/dL) and Low TG (TG < 130 mg/dL). ^**^p < 0.05, calculated using the chi-square test.

HWP was observed in 17 participants, all within the vitamin D deficiency group, while no cases were found in the non-deficiency group (*p* = 0.015). Among its components, vitamin D deficiency was significantly more prevalent in participants with abdominal obesity than in those with low WC, whereas no significant difference was observed between high and low TG groups (Fig 1). Sensitivity analyses were performed using alternative HWP criteria. The association between vitamin D status and HWP remained significant even when the WC threshold was increased to the 90th percentile or the TG cutoff was lowered to 90 mg/dL. Similarly, when vitamin D levels were categorized into three groups (non-deficiency, deficiency, and severe deficiency), a significant association and trend with HWP remained (*p* for trend < 0.05) (S1 and S2 Fig).

### Logistic regression analysis of vitamin D status and HWP

Participants were categorized into deficiency and non-deficiency groups, with deficiency group as the reference. The non-deficiency group had a 12-fold (1/0.082) lower risk of HWP than the deficiency group, though not statistically significant. After adjustment, the risk further decreased to 11-fold (1/0.088) lower, with borderline significance (*p* = 0.060). Among HWP components, the risk of abdominal obesity was lower in the non-deficiency group, with borderline significance (*p* = 0.065) (Table 2). In sensitivity analyses, lowering the TG threshold to 90 mg/dL made the association between vitamin D status and HWP risk statistically significant (*p* = 0.030) (S2 Table). Additionally, when vitamin D status was classified into three groups (severe deficiency, deficiency, and non-deficiency), the non-deficiency group had a significantly lower HWP risk than the severe deficiency group, with a significant trend indicating decreasing risk as vitamin D levels increased (*p* for trend = 0.015) (S3 Table).

**Table 2:** Association Between Vitamin D Status and Hypertriglyceridemic Waist Phenotype (HWP).

Variables	Vitamin D status	Crude model			Adjusted model^a^		
		OR	95% CI	*p*-value	OR	95% CI	*p*-value
HWP	Deficiency (n = 181)	1.00		0.085	1.00		0.060
	Non-Deficiency (n = 57)	0.082	0.01-1.42		0.088	0.01-1.11	
Abdominal obesity	Deficiency (n = 181)	1.00		0.028	1.00		0.065
	Non-Deficiency (n = 57)	0.419	0.19-0.91		0.458	0.19-1.09	
High TG	Deficiency (n = 181)	1.00		0.208	1.00		0.310
	Non-Deficiency (n = 57)	0.447	0.13-1.56		0.640	0.28-1.50	

OR, Odds Ratio; 95% CI, 95% Confidence Interval; HWP, Hypertriglyceridemic Waist Phenotype; TG, Triglycerides. HWP was defined as WC ≥ 75th percentile and TG ≥ 130 mg/dL. Based on these criteria, abdominal obesity was defined as WC ≥ 75th percentile, and high TG was defined as TG ≥ 130 mg/dL. Vitamin D status was categorized as deficiency (<20 ng/mL) and non-deficiency (≥20 ng/mL).

^a^Adjusted for sex, monthly household income, moderate physical activity, total energy intake, follow-up month and supplement use at the age of 13-15 years.

### Comparison of Visceral Fat Indices by Vitamin D Status

**Fig 2:**
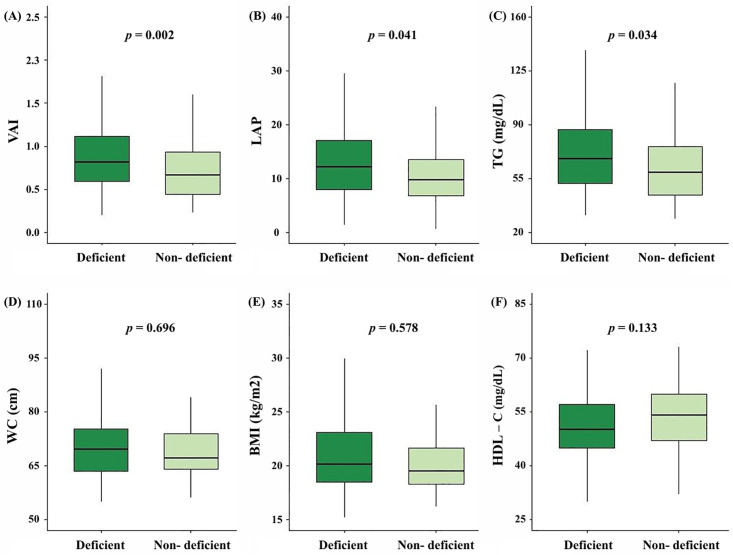
Comparison of Visceral Adiposity Index (VAI), Lipid Accumulation Product (LAP), and Related Variables by Vitamin D Status (A) VAI, (B) LAP, (C) TG, (D) WC, (E) BMI, (F) HDL-C. VAI, Visceral Adiposity Index; LAP, Lipid Accumulation Product; TG, Triglyceride; WC, Waist circumference; BMI, Body mass index; HDL-C, High-density lipoprotein cholesterol. Boxplots show the distribution of each variable by vitamin D status (Deficiency vs. Non-deficiency). The bold line inside each box represents the median, and the whiskers extend to the minimum and maximum values within 1.5 × IQR. *p* values were obtained using the Mann–Whitney U test for non-normally distributed variables (VAI, LAP, TG) and the independent t-test for normally distributed variables (WC, BMI, HDL-C).

Differences in the distribution of visceral fat indicators were observed according to vitamin D status. The deficiency group showed significantly higher median values of VAI (0.85 vs. 0.67, *p* = 0.002), LAP (12.50 vs. 9.76, *p* = 0.041), and TG (68.0 vs. 59.0, *p* = 0.034) compared to the non-deficiency group (Fig 2).

### Linear regression analysis of vitamin D levels and VAI/LAP

Linear regression analysis identified significant inverse associations of vitamin D concentration with log-transformed VAI, LAP, and TG (log VAI: *p* < 0.001; log LAP: *p* = 0.019; log TG: *p* = 0.008). After adjustment, the associations remained significant for log VAI (*p* = 0.008) and log TG (*p* = 0.015), while the association with log LAP showed borderline significance (*p* = 0.060). In contrast, no significant associations were observed for WC, BMI, or HDL-C (Table 3).

**Table 3:** Linear association Between Vitamin D Concentration and Visceral Adiposity Index (VAI) and Lipid Accumulation Product (LAP).

Variables	Crude model			Adjusted model^a^		
	*β*	SE	*p*-value	*β*	SE	*p*-value
log VAI	−0.029	0.007	<0.001	−0.020	0.007	0.008
log LAP	−0.022	0.009	0.019	−0.018	0.010	0.060
log TG^b^	−0.015	0.005	0.008	−0.014	0.006	0.015
Waist circumference (cm)	−0.049	0.115	0.669	−0.174	0.120	0.147
BMI (kg/m^2^)	−0.019	0.043	0.658	−0.029	0.046	0.525
HDL-C (mg/dL)	0.205	0.125	0.102	0.167	0.134	0.216

β, beta coefficient; SE, standard error; log VAI, log-transformed visceral adiposity index; log LAP, log-transformed lipid accumulation product; log TG, log-transformed triglycerides; BMI, body mass index; HDL-C, high-density lipoprotein cholesterol.

^a^Adjusted for sex, monthly household income, moderate physical activity, total energy intake, follow-up month and supplement use at the age of 13–15 years. ^b^log VAI, log LAP, and log TG were used in the analysis because residuals from the untransformed models did not meet the assumption of normality.

## Discussion

This study identified significant associations between vitamin D levels and visceral fat indices (HWP, VAI, and LAP) in adolescents. Approximately 3 out of 4 participants (76.0%) were categorized as vitamin D deficiency (<20 ng/mL), with 19.3% showing severe deficiency (<12 ng/mL). Additionally, HWP was present in 7.1% of the participants. The findings indicated that adolescents in the non-deficiency group had a lower risk of HWP compared to those in the deficiency group. Additionally, higher vitamin D levels were inversely associated with VAI and LAP, suggesting a potential role of vitamin D in visceral fat regulation. These associations remained consistent even after excluding the three participants classified as vitamin D sufficient (≥30 ng/mL) (data not shown).

Previous studies have also reported associations between vitamin D and HWP. A study in Brazilian children aged 6–11 years found that vitamin D deficiency was associated with a 121% higher risk of HWP compared to sufficiency [33]. Similarly, a study in Iranian adults reported a 4-fold higher risk of HWP among vitamin D-deficiency individuals [23], and a study in Chinese patients with type 2 diabetes showed a nearly 6-fold higher HWP risk in the vitamin D-deficiency group [35]. Studies on the association of vitamin D with VAI and LAP have reported conflicting results. A study in Iranian adults found no significant association between vitamin D and VAI, but higher vitamin D levels were associated with a 2.07-fold higher risk of having a high LAP, showing a trend opposite to our findings [24]. In contrast, a study in Italian adults with type 2 diabetes and obesity found that higher vitamin D levels were associated with lower LAP in both groups [25]. Furthermore, a study in Iranian adults with metabolic syndrome reported that vitamin D insufficiency was linked to significantly higher levels of AIP and LAP, although no significant association was found with VAI [36]. Similarly, a study in healthy Korean adults found that higher vitamin D levels were significantly associated with lower visceral fat area (VFA), which aligns with our findings and supports a potential role of vitamin D in visceral fat reduction [37]. Additionally, a study in Brazil investigated the effects of vitamin D supplementation on visceral fat. Obese patients who underwent Roux-en-Y gastric bypass received either 800 IU/day or 1800 IU/day of vitamin D3 for 12 months, with the higher-dose group showing a significantly greater reduction in VAI (*p* = 0.001), indicating that vitamin D supplementation may contribute to visceral fat reduction [38].

Globally, vitamin D deficiency is recognized as a critical public health issue, with adolescents exhibiting the highest prevalence among all age groups. Despite the expectation of greater sun exposure during this life stage, vitamin D deficiency remains highly prevalent in children and adolescents worldwide, including Korea, where nearly 80% are affected [5,6]. Given the role of vitamin D in cardiometabolic health, its deficiency is a concern, with studies linking it to cardiovascular risk in children [39] and to metabolic syndrome in youth populations [40]. In particular, a study of adolescents and young adults at elevated risk of metabolic syndrome reported that low vitamin D levels were significantly associated with metabolic syndrome [40]. In parallel with widespread vitamin D deficiency, the prevalence of obesity, abdominal obesity, hypertension, and metabolic syndrome is rising among children and adolescents. These conditions increase the risk of insulin resistance, dyslipidemia, and cardiovascular diseases, often persisting into adulthood [8–10]. In this context, identifying modifiable factors to mitigate these risks is crucial, with vitamin D emerging as a potential candidate.

Vitamin D plays a role in multiple metabolic pathways that regulate visceral fat accumulation and obesity, which are major contributors to metabolic syndrome and cardiovascular disease. Visceral fat promotes chronic inflammation by increasing pro-inflammatory cytokines while decreasing anti-inflammatory cytokines, contributing to atherosclerosis and metabolic dysfunction. It also exacerbates insulin resistance and elevates triglyceride levels, thereby increasing cardiometabolic risk. Vitamin D may counteract these effects through multiple mechanisms, including promoting energy expenditure, reducing fat storage, and modulating inflammation and oxidative stress. Additionally, it influences lipid metabolism by lowering triglycerides and LDL-C while increasing HDL-C, potentially reducing the risk of cardiometabolic diseases. However, obesity itself may also contribute to vitamin D deficiency due to reduced physical activity and the sequestration of vitamin D in excess fat tissue. The precise mechanisms linking vitamin D, visceral fat, and obesity-related health risks remain unclear, warranting further investigation [12–13].

Vitamin D has a protective effect cardiometabolic health [41,42], and this study further supports this association. A significant relationship was observed between vitamin D levels and visceral fat indices, suggesting its potential role in cardiometabolic regulation. Despite data collection between April and August—outside the winter season when vitamin D levels are typically lower—76% of adolescents were vitamin D deficiency, and 98.7% had insufficient or deficiency levels (<30 ng/mL). This underscores the persistence of vitamin D deficiency even during adolescence, a life stage characterized by relatively high physical activity and sun exposure, emphasizing the necessity of targeted interventions. Consistently, a KNHANES-based study reported that nearly 80% of Korean adolescents had vitamin D deficiency (<20 ng/mL), with lifestyle factors such as limited outdoor activity highlighted as major contributors [43]. With the rising prevalence of young-onset obesity and visceral fat accumulation, cardiometabolic health concerns are escalating. Given vitamin D’s potential role in mitigating these risks, strategies such as promoting outdoor activity, improving dietary intake, and considering supplementation may be essential for supporting adolescent cardiometabolic health and mitigating the long-term burden of cardiometabolic diseases. Nutrition and broader dietary patterns have been shown to play a role in supporting cardiometabolic health [44–45]. However, research specifically focusing on vitamin D within these patterns remains insufficient, and further studies considering multiple influencing factors are needed.

This study has several limitations. As it is based on a birth cohort from a single hospital, the findings may not be generalizable to all adolescents. Potential information bias from measurement and survey responses could also affect the results. Additionally, the absence of body composition analysis prevented direct assessment of visceral adipose tissue (VAT), relying instead on indirect indices. As a cross-sectional study, it cannot establish causality and does not fully account for seasonal variations and other external factors. Finally, because the number of HWP events was small (7.1%), the post-hoc power for the logistic regression was limited (61.4%), indicating that larger-scale studies are needed to more reliably estimate odds ratios for this outcome.

Despite these limitations, this study is the first to investigate the relationship between vitamin D and visceral fat indices in adolescents. By integrating HWP, VAI, and LAP, this study provides a useful assessment of cardiometabolic health, with sensitivity analyses further strengthening the reliability of the findings. These results contribute to understanding adolescent cardiometabolic health and may inform preventive strategies to reduce long-term disease risk.

In conclusion, we found a significant association between vitamin D levels and visceral fat indices (HWP, VAI, and LAP) in adolescents, suggesting a potential role of vitamin D in cardiometabolic health. Given the high prevalence of vitamin D deficiency and the rising concern of young-onset obesity, assessing vitamin D levels in adolescents may be warranted to prevent cardiometabolic risk

## Supporting information

S1 Fig Distribution of HWP by Criteria and Vitamin D Status (Two Groups). HWP, Hypertriglyceridemic Waist Phenotype; The criteria for HWP are as follows – HWP 1: Waist circumference (WC) ≥75th percentile and triglycerides (TG) ≥130 mg/dL. HWP 2: WC ≥ 75th percentile and TG ≥ 90 mg/dL. HWP 3: WC ≥ 90th percentile and TG ≥ 130 mg/dL. Vitamin D status was categorized as Deficiency (<20 ng/mL) and Non-Deficiency (≥20 ng/mL). ^*^p-values are calculated using chi-square test.
(TIF)

S2 Fig Distribution of HWP by Criteria and Vitamin D Status (Three Groups). HWP, Hypertriglyceridemic Waist Phenotype; The criteria for HWP are as follows – HWP 1: Waist circumference (WC) ≥75th percentile and triglycerides (TG) ≥130 mg/dL. HWP 2: WC ≥ 75th percentile and TG ≥ 90 mg/dL. HWP 3: WC ≥ 90th percentile and TG ≥ 130 mg/dL. Vitamin D status was categorized as Severe Deficiency (<12 ng/mL), Deficiency (12–19 ng/mL), and Non-Deficiency (≥20 ng/mL). ^*^p-values are calculated using chi-square test ^†^*p* for trend values are calculated using the Cochran-Armitage trend test to assess the trend across vitamin D categories.
(TIF)

S1 Table Distribution of covariates across vitamin D deficiency and non-deficiency groups. Serum 25-hydroxyvitamin D [25(OH)D] was categorized as Non-deficiency (≥20 ng/mL) and Deficiency (<20 ng/mL). a Continuous variables were analyzed using the t-test. Categorical variables were analyzed using the chi-square test.
(DOCX)

S2 Table Logistic Regression Analysis of the Association Between HWP and Vitamin D Status (Two Groups). HWP, Hypertriglyceridemic Waist Phenotype; OR, Odds Ratio; 95% CI, 95% Confidence Interval. ^a^The criteria for HWP are as follows – HWP 1: Waist circumference (WC) ≥75th percentile and triglycerides (TG) ≥130 mg/dL (n = 17). HWP 2: WC ≥ 75th percentile and TG ≥ 90 mg/dL (n = 30). HWP 3: WC ≥ 90th percentile and TG ≥ 130 mg/dL (n = 8). ^b^Vitamin D status was categorized as Deficiency (<20 ng/mL) and Non-Deficiency (≥20 ng/mL). ^c^Adjusted for sex, monthly household income, moderate physical activity, total energy intake, and supplement use at the age of 13–15 years.
(DOCX)

S3 Table Logistic Regression Analysis of the Association Between HWP and Vitamin D Status (Three Groups). HWP, Hypertriglyceridemic Waist Phenotype; OR, Odds Ratio; 95% CI, 95% Confidence Interval. ^a^The criteria for HWP are as follows – HWP 1: Waist circumference (WC) ≥75th percentile and triglycerides (TG) ≥130 mg/dL (n = 17). HWP 2: WC ≥ 75th percentile and TG ≥ 90 mg/dL (n = 30). HWP 3: WC ≥ 90th percentile and TG ≥ 130 mg/dL (n = 8). ^b^Vitamin D status was categorized as Severe Deficiency (<12 ng/mL), Deficiency (12–19 ng/mL), and Non-Deficiency (≥20 ng/mL). ^c^ Adjusted for sex, monthly household income, moderate physical activity, average energy intake, and supplement use at the age of 13–15 years.
(DOCX)
