# Supplementary figures and images for: Association between serum vitamin D deficiency and visceral fat indices in adolescents: The Ewha Birth and growth cohort study
Source: PLoS One. 2025 Oct 31;20(10):e0335507. doi: 10.1371/journal.pone.0335507 (PMC12578238; doi:10.1371/journal.pone.0335507)

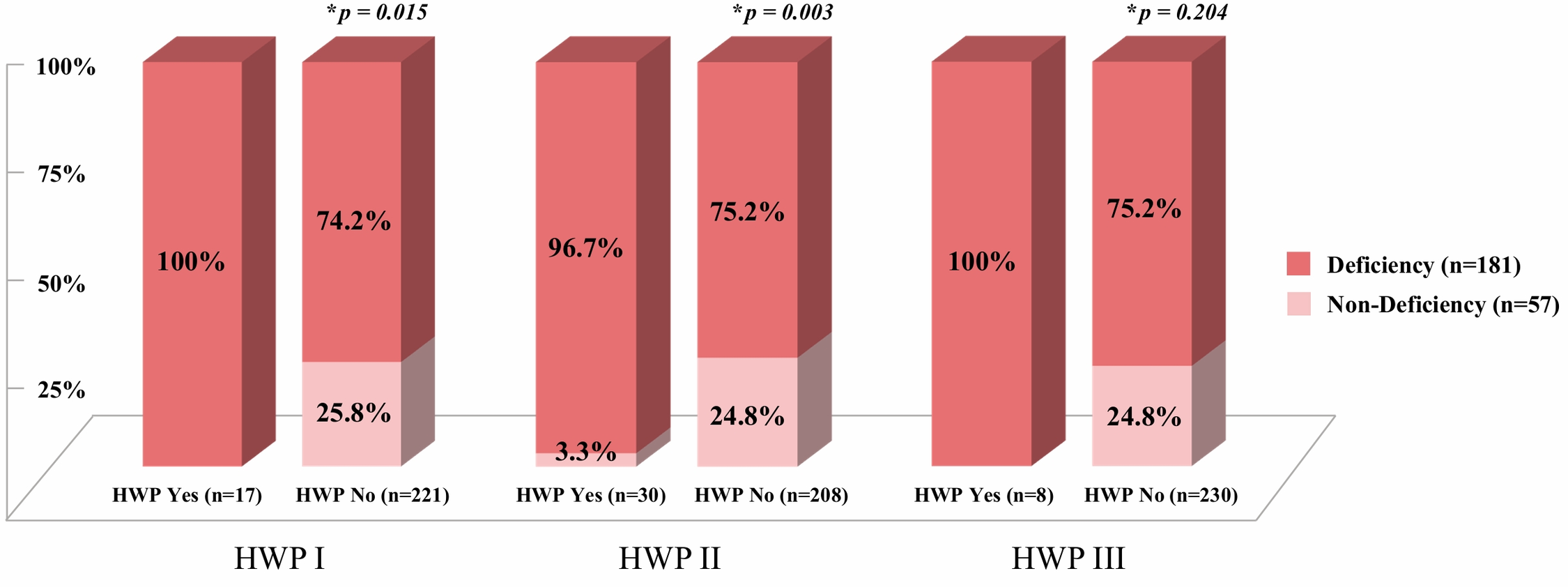

Supplement: S1 Fig — HWP, Hypertriglyceridemic Waist Phenotype; The criteria for HWP are as follows – HWP 1: Waist circumference (WC) ≥75th percentile and triglycerides (TG) ≥130 mg/dL. HWP 2: WC ≥ 75th percentile and TG ≥ 90 mg/dL. HWP 3: WC ≥ 90th percentile and TG ≥ 130 mg/dL. Vitamin D status was categorized as Deficiency (<20 ng/mL) and Non-Deficiency (≥20 ng/mL). *p-values are calculated using chi-square test. (TIF) [file pone.0335507.s001.tif]

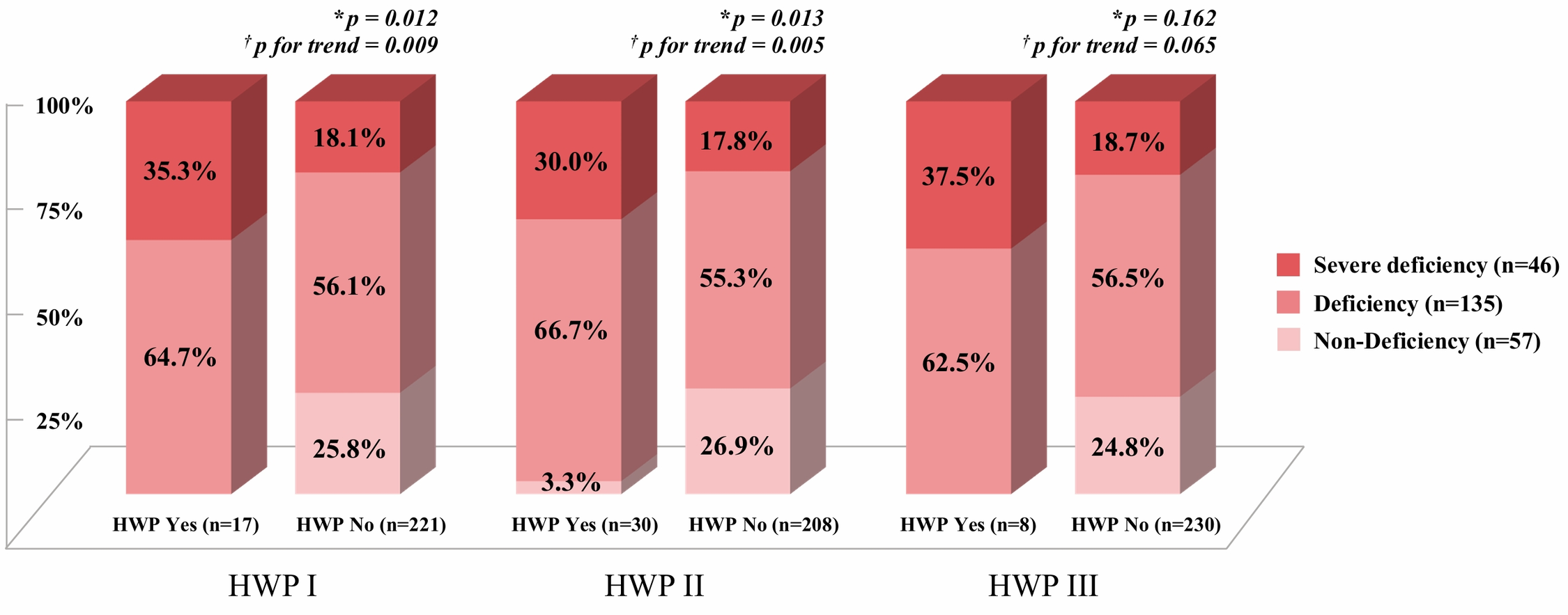

Supplement: S2 Fig — HWP, Hypertriglyceridemic Waist Phenotype; The criteria for HWP are as follows – HWP 1: Waist circumference (WC) ≥75th percentile and triglycerides (TG) ≥130 mg/dL. HWP 2: WC ≥ 75th percentile and TG ≥ 90 mg/dL. HWP 3: WC ≥ 90th percentile and TG ≥ 130 mg/dL. Vitamin D status was categorized as Severe Deficiency (<12 ng/mL), Deficiency (12–19 ng/mL), and Non-Deficiency (≥20 ng/mL). *p-values are calculated using chi-square test †p for trend values are calculated using the Cochran-Armitage trend test to assess the trend across vitamin D categories. (TIF) [file pone.0335507.s002.tif]
