# Supplementary material for: Association between serum vitamin D deficiency and visceral fat indices in adolescents: The Ewha Birth and growth cohort study
Source: PLoS One. 2025 Oct 31;20(10):e0335507. doi: 10.1371/journal.pone.0335507 (PMC12578238; doi:10.1371/journal.pone.0335507)
Supplement: S1 Table — Serum 25-hydroxyvitamin D [25(OH)D] was categorized as Non-deficiency (≥20 ng/mL) and Deficiency (<20 ng/mL). a Continuous variables were analyzed using the t-test. Categorical variables were analyzed using the chi-square test. (DOCX) [file pone.0335507.s003.docx]

| **S1 Table. Distribution of covariates across vitamin D deficiency and non-deficiency groups** | | | | |
| --- | --- | --- | --- | --- |
| Variables | Total  (n=238) | Deficiency (n=181) | Non-Deficiency (n=57) | *p-*value^a^ |
| Monthly household income | | | | |
| < 3 million KRW, n (%) | 16 (6.9) | 11 (6.2) | 5 (8.9) | 0.737 |
| 3–5 million KRW, n (%) | 68 (29.2) | 51 (28.8) | 17 (30.4) |  |
| ≥5 million KRW, n (%) | 149 (63.9) | 115 (65.0) | 34 (60.7) |  |
| Moderate physical activity | | | | |
| Never, n (%) | 44 (18.7) | 40 (22.3) | 4 (7.1) | 0.023 |
| 1~2 times/week, n (%) | 103 (43.8) | 78 (43.6) | 25 (44.7) |  |
| ≥3 times/week, n (%) | 88 (37.5) | 61 (34.1) | 27 (48.2) |  |
| Growth-related dietary supplements | | | | |
| Yes, n (%) | 49 (2.7) | 32 (17.8) | 17 (29.8) | 0.050 |
| No, n (%) | 188 (79.3) | 148 (82.2) | 40 (70.2) |  |
| Follow-up month | | | | |
| April, n (%) | 38 (16.0) | 31 (17.1) | 7 (12.3) | 0.064 |
| May, n (%) | 169 (71.0) | 122 (67.4) | 47 (82.5) |  |
| July-August, n (%) | 31 (13.0) | 28 (15.5) | 3 (5.3) |  |
| Total energy intake (kcal/day) | 2116.44±577.44 | 2106.65±583.99 | 2147.55 ±560.04 | 0.642 |
| Serum 25-hydroxyvitamin D [25(OH)D] was categorized as Non-deficiency (≥20 ng/mL) and Deficiency (<20 ng/mL). ^a^ Continuous variables were analyzed using the t-test, and categorical variables were analyzed using the chi-square test.. | | | | |
