# Supplementary material for: Association between serum vitamin D deficiency and visceral fat indices in adolescents: The Ewha Birth and growth cohort study
Source: PLoS One. 2025 Oct 31;20(10):e0335507. doi: 10.1371/journal.pone.0335507 (PMC12578238; doi:10.1371/journal.pone.0335507)
Supplement: S2 Table — HWP, Hypertriglyceridemic Waist Phenotype; OR, Odds Ratio; 95% CI, 95% Confidence Interval. aThe criteria for HWP are as follows – HWP 1: Waist circumference (WC) ≥75th percentile and triglycerides (TG) ≥130 mg/dL (n = 17). HWP 2: WC ≥ 75th percentile and TG ≥ 90 mg/dL (n = 30). HWP 3: WC ≥ 90th percentile and TG ≥ 130 mg/dL (n = 8). bVitamin D status was categorized as Deficiency (<20 ng/mL) and Non-Deficiency (≥20 ng/mL). cAdjusted for sex, monthly household income, moderate physical activity, total energy intake, and supplement use at the age of 13–15 years. (DOCX) [file pone.0335507.s004.docx]

| **S2 Table. Logistic Regression Analysis of the Association Between HWP and Vitamin D Status (Two Groups)** | | | | | | | | |
| --- | --- | --- | --- | --- | --- | --- | --- | --- |
| Criteria^a^ | Vitamin D status^b^ | Crude model | | | Adjusted model^c^ | | | |
|  |  | OR | 95% CI | *p*-value | OR | 95% CI | *p*-value |  |
| HWP1 | Deficiency (n=181) | 1.00 |  | 0.085 | 1.00 |  | 0.060 |  |
|  | Non-Deficiency (n=57) | 0.082 | 0.00-1.42 |  | 0.088 | 0.01-1.11 |  |  |
| HWP2 | Deficiency (n=181) | 1.00 |  | 0.021 | 1.00 |  | 0.030 |  |
|  | Non-Deficiency (n=57) | 0.094 | 0.01-0.70 |  | 0.167 | 0.03-0.84 |  |  |
| HWP3 | Deficiency (n=181) | 1.00 |  | 0.241 | 1.00 |  | 0.121 |  |
|  | Non-Deficiency (n=57) | 0.177 | 0.01-3.20 |  | 0.143 | 0.01-1.67 |  |  |
| HWP, Hypertriglyceridemic Waist Phenotype; OR, Odds Ratio; 95% CI, 95% Confidence Interval. ^a^The criteria for HWP are as follows - HWP 1: Waist circumference (WC) ≥75th percentile and triglycerides (TG) ≥130 mg/dL (n=17). HWP 2: WC ≥75th percentile and TG ≥90 mg/dL (n=30). HWP 3: WC ≥90th percentile and TG ≥130 mg/dL (n=8). ^b^Vitamin D status was categorized as Deficiency (<20 ng/mL) and Non-Deficiency (≥20 ng/mL). ^c^Adjusted for sex, monthly household income, moderate physical activity, total energy intake, follow-up month and supplement use at the age of 13-15 years. | | | | | | | | |
