# Supplementary material for: Association between serum vitamin D deficiency and visceral fat indices in adolescents: The Ewha Birth and growth cohort study
Source: PLoS One. 2025 Oct 31;20(10):e0335507. doi: 10.1371/journal.pone.0335507 (PMC12578238; doi:10.1371/journal.pone.0335507)
Supplement: S3 Table — HWP, Hypertriglyceridemic Waist Phenotype; OR, Odds Ratio; 95% CI, 95% Confidence Interval. aThe criteria for HWP are as follows – HWP 1: Waist circumference (WC) ≥75th percentile and triglycerides (TG) ≥130 mg/dL (n = 17). HWP 2: WC ≥ 75th percentile and TG ≥ 90 mg/dL (n = 30). HWP 3: WC ≥ 90th percentile and TG ≥ 130 mg/dL (n = 8). bVitamin D status was categorized as Severe Deficiency (<12 ng/mL), Deficiency (12–19 ng/mL), and Non-Deficiency (≥20 ng/mL). c Adjusted for sex, monthly household income, moderate physical activity, average energy intake, and supplement use at the age of 13–15 years. (DOCX) [file pone.0335507.s005.docx]

| **S3 Table. Logistic Regression Analysis of the Association Between HWP and Vitamin D Status (Three Groups)** | | | | | | | | | | |
| --- | --- | --- | --- | --- | --- | --- | --- | --- | --- | --- |
| Criteria^a^ | Vitamin D status^b^ | Crude model | | | | Adjusted model^c^ | | | |  |
|  |  | OR | 95% CI | *p*-value | *p* for trend | OR | 95% CI | *p*-value | *p for trend* |  |
| HWP1 | Severe deficiency (n=46) | 1.00 |  |  | 0.011 | 1.00 |  |  | 0.015 |  |
|  | Deficiency (n=135) | 0.576 | 0.21-1.62 | 0.295 |  | 0.583 | 0.20-1.69 | 0.319 |  |  |
|  | Non-Deficiency (n=57) | 0.054 | 0.00-1.01 | 0.051 |  | 0.057 | 0.00-0.82 | 0.035 |  |  |
| HWP2 | Severe deficiency (n=46) | 1.00 |  |  | 0.006 | 1.00 |  |  | 0.036 |  |
|  | Deficiency (n=135) | 0.715 | 0.30-1.71 | 0.450 |  | 0.843 | 0.33-2.13 | 0.718 |  |  |
|  | Non-Deficiency (n=57) | 0.073 | 0.01-0.60 | 0.015 |  | 0.145 | 0.02-0.85 | 0.033 |  |  |
| HWP3 | Severe deficiency (n=46) | 1.00 |  |  | 0.073 | 1.00 |  |  | 0.050 |  |
|  | Deficiency (n=135) | 0.524 | 0.13-2.11 | 0.363 |  | 0.536 | 0.14-2.04 | 0.361 |  |  |
|  | Non-Deficiency (n=57) | 0.108 | 0.01-2.21 | 0.148 |  | 0.086 | 0.01-1.23 | 0.070 |  |  |
| HWP, Hypertriglyceridemic Waist Phenotype; OR, Odds Ratio; 95% CI, 95% Confidence Interval. ^a^The criteria for HWP are as follows - HWP 1: Waist circumference (WC) ≥75th percentile and triglycerides (TG) ≥130 mg/dL (n=17). HWP 2: WC ≥75th percentile and TG ≥90 mg/dL (n=30). HWP 3: WC ≥90th percentile and TG ≥130 mg/dL (n=8). ^b^Vitamin D status was categorized as Severe Deficiency (<12 ng/mL), Deficiency (12–19 ng/mL), and Non-Deficiency (≥20 ng/mL). ^c^ Adjusted for sex, monthly household income, moderate physical activity, total energy intake, follow-up month and supplement use at the age of 13-15 years. | | | | | | | | | | |
